# Effect on Germline Mutation Rate in a High-Risk Chinese Breast Cancer Cohort after Compliance with The National Comprehensive Cancer Network (NCCN) 2023 v.1 Testing Criteria

**DOI:** 10.3390/cancers15092635

**Published:** 2023-05-06

**Authors:** Ava Kwong, Cecilia Y. S. Ho, Wing-Pan Luk, Ling-Hiu Fung, Chun-Hang Au, Edmond S. K. Ma

**Affiliations:** 1 Division of Breast Surgery, Department of Surgery, The University of Hong Kong, Hong Kong SAR, China; 2 Hong Kong Hereditary Breast Cancer Family Registry, Hong Kong SAR, China; 3 Department of Surgery, Hong Kong Sanatorium & Hospital, Hong Kong SAR, China; 4 Division of Molecular Pathology, Department of Pathology, Hong Kong Sanatorium & Hospital, Hong Kong SAR, China; 5 Department of Research, Hong Kong Sanatorium & Hospital, Hong Kong SAR, China

**Keywords:** hereditary breast cancers, Chinese, germline mutation, NCCN

## Abstract

**Simple Summary:**

The NCCN Clinical Practice Guidelines are testing criteria used to identify high-risk individuals associated with an increased risk of breast, ovarian, pancreatic, and prostate cancer. The testing criteria have been recently modified in early 2023. In this study, we provided a real-world application of the updated criteria and their effect on germline mutation rates in Chinese high-risk breast cancer patients. An additional 6.4% (242/3797) of the patients were included after the revision; the mutation rates in these newly included patients were 2.1% and 2.5% for *BRCA1/2* and all six high-penetrance genes (*BRCA1*, *BRCA2*, *CDH1*, *PALB2*, *PTEN*, and *TP53*), respectively. Applying the updated criteria for genetic investigation would increase the number of positive detection, leading to potentially more patients. However, the balance between the resource and benefits requires further consideration.

**Abstract:**

Background: The National Comprehensive Cancer Network (NCCN) testing criteria for the high-penetrance breast cancer susceptibility genes, specifically *BRCA1*, *BRCA2*, *CDH1*, *PALB2*, *PTEN*, and *TP53*, have been recently modified in 2023 to 2023 v.1. The following criteria have been changed: (1) from a person diagnosed with breast cancer at ≤45 to ≤50; (2) from aged 45–50 of personal breast diagnosis to any age of diagnosis with multiple breast cancers; and (3) from aged ≥51 of personal breast diagnosis to any age of diagnosis with family history listed in NCCN 2022 v.2. Methods: High-risk breast cancer patients (*n* = 3797) were recruited from the Hong Kong Hereditary Breast Cancer Family Registry between 2007 and 2022. Patients were grouped according to NCCN testing criteria 2023 v.1 and 2022 v.2. A 30-gene panel for hereditary breast cancer was performed. The mutation rates on high-penetrance breast cancer susceptibility genes were compared. Results: About 91.2% of the patients met the 2022 v.2 criteria, while 97.5% of the patients met the 2023 v.1 criteria. An extra 6.4% of the patients were included after the revision of the criteria, and 2.5% of the patients did not meet both testing criteria. The germline *BRCA1/2* mutation rates for patients meeting the 2022 v.2 and 2023 v.1 criteria were 10.1% and 9.6%, respectively. The germline mutation rates of all 6 high-penetrance genes in these two groups were 12.2% and 11.6%, respectively. Among the additional 242 patients who were included using the new selection criteria, the mutation rates were 2.1% and 2.5% for *BRCA1/2* and all 6 high-penetrance genes, respectively. Patients who did not meet both testing criteria were those with multiple personal cancers, a strong family history of cancers not listed in the NCCN, unclear pathology information, or the patient’s voluntary intention to be tested. The mutation rates of *BRCA1/2* and the 6 high-penetrance genes in these patients were 5.3% and 6.4%, respectively. Conclusion: This study provided a real-world application of the revision of NCCN guidelines and its effect on the germline mutation rate in the Chinese population. Applying the updated criteria for further genetic investigation would increase the positive detection rate, and potentially more patients would benefit. The balance between the resource and outcome requires careful consideration.

## 1. Introduction

Hereditary cancers are often caused by pathogenic or likely pathogenic (P/LP) variants in genes involved in regulating cell growth and/or DNA repair [1,2,3]. P/LP variants in these genes are often associated with increased risk for certain cancers (i.e., breast, ovarian, prostate, colon, and pancreatic cancers) with an early onset and exhibit an autosomal dominant inheritance pattern [4,5]. Assessment of an individual’s risk for hereditary cancer is based on a thorough evaluation of the personal and family history. For hereditary cancers, advances in molecular genetics have identified several high- to moderate-penetrance genes associated with inherited susceptibility to breast, ovarian, and pancreatic cancers (e.g., *BRCA1*, *BRCA2*, *CDH1*, *PALB2*, *PTEN*, and *TP53*) [6,7,8]. The NCCN Clinical Practice Guidelines in Oncology for Genetic/Familial High-Risk Assessment: Breast, Ovarian, and Pancreatic has been established for over 20 years, which included the testing criteria to identify high-risk individuals associated with increased risk of breast, ovarian, pancreatic, and prostate cancer [9,10]. Current testing guidelines primarily focus on *BRCA1*, *BRCA2*, *CDH1*, *PALB2*, *PTEN*, and *TP53*. This guideline helped select patients with the highest likelihood of mutation carriers and increased the cost–benefit ratio of genetic testing and subsequent clinical management [11]. With the development of next-generation sequencing (NGS), the availability and cost of testing have been reduced [8]. King and co-workers were the first group advocating population-based germline *BRCA1/2* screening for all women in 2014 [12], but it was met with controversy. The American College of Medical Genetics and Genomics (ACMG) also suggested to subsequently evaluate the need for germline genetic testing on all patients with breast cancer for hereditary breast cancer [13]. The American Society of Breast Surgeons published a consensus statement with similar recommendations in 2019 [14]. However, in 2023, the NCCN reviewed their testing guidelines and decided to remain limiting the germline genetic testing on high-risk hereditary breast and ovarian cancer (HBOC) patients for *BRCA1*, *BRCA2*, *CDH1*, *PALB2*, *PTEN*, and *TP53* genes only instead of a universal testing on all patients with breast cancer. The NCCN panel favored a confined approach due to the low positive detection rate when a large screening panel of genes was tested on all breast cancer patients. There was also a lack of evidence-based input for rarer genes to support risk management. Appropriate use of resources is another consideration. There was a shortage of well-trained genetics counselor and health professionals to provide genetic counseling and the subsequence spinout procedures, such as surveillance cancer screenings, prophylactic surgeries and caring, genetics counseling on related family members, and reproductive planning supports. These situations were not just locally happening in Hong Kong. There were, in fact, a worldwide issue applicable to any country providing or planning to provide such a genetic service. Here, we provided a review and re-examination in our Hong Kong high-risk breast cohort, evaluating the increase in the number of tested patients and the expansions on the spectrum of mutation carriers in the additional actionable genetic variants found by multigene panel testing after compliance with the most current NCCN guideline when compared with the previous version of the NCCN eligibility criteria.

## 2. Materials and Methods

### 2.1. Participants and Selection Criteria

A total of 3797 Asian individuals were recruited through the Hong Kong Hereditary Breast Cancer Family Registry from March 2007 to August 2022 for germline mutation screening. Patients were eligible to participate if they fulfilled the following selection criteria: (1) diagnosed with breast cancer with at least one first- or second-degree relative with breast and/or ovarian cancer, regardless of age; (2) diagnosed with breast cancer ≤50 years; (3) had bilateral breast cancer; (4) had triple-negative breast cancer; (5) male breast cancer; and (6) had personal breast cancer and other cancer. Informed consent was obtained from all recruited participants, and the research was conducted according to the Declaration of Helsinki.

### 2.2. DNA Extraction and Sequencing

Genomic DNA extraction from peripheral blood was performed using a QIAamp DNA Blood Mini Kit (Qiagen, Hilden, Germany) or a QIAsymphony DNA Mini Kit (Qiagen, Hilden, Germany) according to the manufacturer’s instructions. Qualified DNA was pooled and sequenced with a 30-gene panel (Color Genomics Laboratory, Burlingame, CA, USA) or a 93-gene DHS-001Z human breast cancer panel (Qiagen, Hilden, Germany) on MiSeq or NextSeq (Illumina, San Diego, CA, USA) instruments. The minimum sequencing depth and median coverage were typically 50-fold of 200–300×. All detected pathogenic variants were further validated by conventional Sanger bi-directional DNA sequencing.

### 2.3. Variant Interpretation and Annotation

Variant calling bioinformatics was performed as previously described [15,16]. Paired sequencing reads were mapped to human reference genome sequence GRCh37/hg19. Variants with a minor allele frequency of at least 1% reported by The 1000 Genomes Projects [17] were excluded from manual variant curation. Variants were described according to the recommendations of the Human Genome Variation Society (HGVS) nomenclature (http://varnomen.hgvs.org/, accessed on 1 February 2023). The variant descriptions were further cross-checked with a Mutalyzer Name Checker (http://mutalyzer.nl, accessed on 1 February 2023).

### 2.4. Statistical Analysis

Fisher’s exact test was used to study the relationship between selection variables and the mutation status. The limit of significance for all analyses was defined as a *p*-value of <0.05. Data analyses were performed using the statistical software R (version 3.4.2) [18].

## 3. Results

### 3.1. Patients’ Characteristics of the Cohorts

Our testing cohort included 3797 patients with breast cancer. All patients underwent germline genetic testing with a panel of at least 30 genes (Supplementary Table S1). The median age at breast cancer diagnosis was 44 years (range 18–95). Bilateral breast cancers were seen in 747 patients (19.7%). Most of the breast cancers were invasive ductal carcinoma (NOS type) (3212, 72.7%). A high percentage of breast cancers were of luminal type (2699, 74.5%). Triple-negative breast cancers (TNBCs) were also common (573, 15.8%); only 8.5% were HER2-positive breast cancers. Most of the breast tumors were diagnosed at early stages (0, I, or II) (3655, 85.4%) and with grading of 2 or 3 (1437, 45.7% and 1146, 36.4%, respectively). There were 305 patients (8%) with multiple personal cancers. A positive family history of breast cancer (first- to third-degree relatives) was seen among 1655 patients (43.6%). Family history of ovarian, prostate, and pancreatic cancer in first- to third-degree relatives were 202 (5.3%), 201 (5.3%), and 186 (4.9%), respectively, of their relatives. Detailed clinicopathological characteristics are shown in Table 1.

### 3.2. NCCN Testing Guideline 2022 v.2 vs. 2023 v.1

There were recent modifications to the NCCN testing criteria for the high-penetrance breast cancer susceptibility genes. Testing was recommended to patients with a personal history of breast cancer at below 45 in 2022 and was amended to below 50. The testing criteria used for patients with a family history of breast, ovarian, pancreatic, or prostate cancer have been relaxed from personal diagnosis at the age 46–50 to any age. Families with more than two family members having breast or prostate cancers at any age were advised for genetic tests without considering the proband’s age of diagnosis in the updated testing criteria. In patients with multiple breast cancers, the testing age was also changed from 46–50 to any age. The details on the changes of testing criteria are listed in Table 2.

### 3.3. Germline Mutation Detection Rate

**Figure 1:**
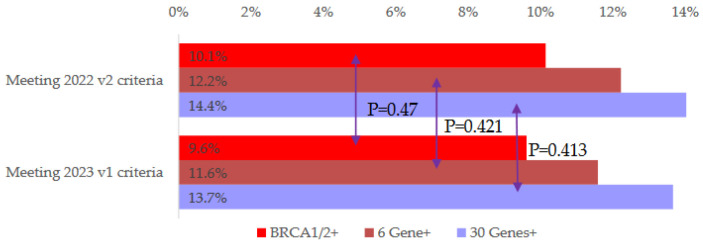
Difference in mutation rate for cohorts fulfilling NCCN 2022 v.2 and 2023 v.1 criteria.

A total of 514 (13.5%) out of 3797 patients had a P/LP mutation variant in 30 genes. By analyzing the pedigrees and respective personal breast cancer clinicopathological characteristics from these patients, they were classified according to the NCCN testing guideline 2022 v.2 and 2023 v.1 criteria. Of these, 3461 (91.2%) of them met the 2022 v.2 criteria, and 3703 (97.5%) met the 2023 v.1 testing criteria. An additional 242 patients (6.4%) met the 2023 v.1 criteria but would be excluded from genetic testing using the 2022 v.2 criteria. Lastly, 94 patients (2.5%) did not meet both testing criteria. The germline *BRCA1/2* mutation rate for patients meeting the 2022 v.2 criteria was 10.1% (351/3461), and the rate for those meeting the 2023 v.1 criteria was 9.6% (356/3703). Of these additional patients who met the 2023 v.1 criteria, the germline *BRCA1/2* mutation rate was 2.1% (5/242). However, the difference in the percentage of the *BRCA1/2* positive rate in these two groups (who met the NCCN testing guideline 2022 v.2 or 2023 v.1 criteria) did not reach statistical significance (*p* = 0.476). The germline mutation rate of the 6 high-penetrance genes (for *BRCA1*, *BRCA2*, *CDH1*, *PALB2*, *PTEN*, and *TP53*) in patients meeting the 2022 v.2 criteria was 12.2% (423/3461), and the rate of those meeting the 2023 v.1 criteria was 11.6% (429/3703); the difference in positive cases between these two groups was not statistically significant (*p* = 0.4218). The mutation rates for patients who did not meet both testing criteria were 5.3% for *BRCA1/2* and 6.4% for all 6 high-penetrance genes. These patients were those with multiple personal cancers, with a strong family history of cancers not listed in the NCCN, with unclear pathology information, or in which tested because of their own intentions. On a 30-gene panel with an elevated risk of hereditary cancers, including breast, ovarian, uterine/endometrial, colorectal, melanoma, pancreatic, prostate, and stomach [12], the mutation rate for patients meeting the 2022 v.2 criteria was 14.4% (497/3461), and the rate for those meeting the 2023 v.1 criteria was 13.7% (506/3703); the difference in positive cases between these two groups was not statistically significant (*p* = 0.4136) and with an extra 3.7% (9/242) detection rate on pathogenic or likely pathogenic mutation variants with the new 2023 NCCN testing guideline. Details of the mutation rate are listed in Figure 1 and Table 3.

### 3.4. Patients who Met 2023 v.1 Criteria Only

Among 242 patients who met the 2023 v.1 criteria only, most of these breast cancer patients were diagnosed at 45–50 (37.4%) and 62% had bilateral breast cancers. A total of 84 patients met the new testing criteria because of their diagnosis age (45–50); the *BRCA1/2* mutation rate was 3.6%, and it was 4.8% in all six high-penetrance genes. The 30-gene mutation rate of this group was 7.1%. One hundred fifty bilateral breast cancer patients with a diagnosis age over 50 were also included in these new criteria. The mutation rate was 1.3% for both *BRCA1/2* and all 6 high-penetrance genes, and the mutation rate for 30 genes was 2%. None of these patients had a family history of breast, ovarian, prostate, or pancreatic cancer. Detailed clinicopathological characteristics of these patients are shown in Table 4.

## 4. Discussion

With the revision of the NCCN testing criteria, an extra 242 (6.4%) patients were included after the revision of the criteria; 91.2% of the patients met the 2022 v.2 criteria, while 97.6% of the patients met the 2023 v.1 criteria, and 2.5% of the patients do not meet both testing criteria. The germline *BRCA1/2* mutation rates for patients meeting the 2022 v.2 and 2023 v.1 criteria were 10.1% and 9.6%, respectively. The germline mutation rates of all 6 high-penetrance genes in these two groups were 12.2% and 11.6%, respectively. The mutation rates for 30 hereditary-cancer-associated genes in these two groups were 14.4% and 13.7%, respectively. With the revision of the NCCN testing criteria, a total of 9 out of 242 (3.7%) mutation carriers were detected. The positive detection rates for *BRCA1/2*, 6 high-penetrance genes, and 30 hereditary-cancer-associated genes were 2.1%, 2.5%, and 3.7%, respectively. This shows that the update is also applicable for the Chinese population. In the old days, when the NCCN testing guideline included *BRCA1/2* only, there was a study from the US that involved 959 patients on an 80-gene multicancer panel; of those patients who met the 2017 NCCN *BRCA1/2* testing guidelines, 9.4% had a P/LP variant. Of those patients who did not meet the 2017 testing guidelines, 7.9% had a P/LP variant. The difference in positive results between these groups was not statistically significant (*p* = 0.424) [19]. Another US study in which the exomes of 50,000 patients were sequenced found that 22.8% of *BRCA1* carriers and 44.9% of *BRCA2* carriers did not meet the 2017 published NCCN testing guidelines [16]. In a study by Buys et al. [20] on 35,000 patients from multiple ancestries with breast cancer, there was a P/LP variant rate of 9.3% for a 25-gene panel, and the positive rate ranged from 7.2% to 11.5% based on ancestry. The mutation rate of our 6 mentioned high-penetrance genes was 5.8%, and more than 50% of these variants were in genes other than *BRCA1/2*. About 9.6% of their women met the NCCN testing criteria and had a mutation identified compared with 5.9% for those who did not meet the criteria. Another study by Susswein et al. [21] reported a consecutive series of 10,000 cancer patients and unaffected individuals on 29-gene NGS testing, with 82% of the patients being Caucasian. Of these, half of the P/LP variants identified in patients with breast or ovarian cancer were in genes other than *BRCA1/2*. The results of a prospective study on postmenopausal women with breast cancer showed that 3.6% of them were harboring a P/LP variant on a panel of 28 breast-cancer-associated genes [22], while in another study on 588 women ≥65 years with breast cancer, the rate for identifying breast-cancer-related P/LP variants was 5.6% [23]. A study on over 3900 women with breast cancer who met or did not meet the NCCN 2020 v.1 criteria confirmed the mutation rate of 9.0% vs. 3.5% on the 9 predisposition genes (*ATM*, *BRCA1*, *BRCA2*, *CDH1*, *CHEK2*, *NF1*, *PALB2*, *PTEN* and *TP53*) [24]. Another multicenter test of an 80-gene panel test on 2984 patients with unselected patient personal cancer history and family history showed that the P/LP variant was found in 13.3%, 5% of the mutations were from a highly penetrant gene, and more than half of the identified variants were of genes with moderate or low penetrance [25]. A similar study on current England NHS test criteria for genetic testing also showed that 4.6% of pathogenic mutation carriers did not fulfill NHS eligibility criteria, all of which were actionable; nearly 1 in 20 individuals required alternation in management [11]. These studies confirmed the need to modify testing guidelines for other moderate- to low-penetrance genes, not only *BRCA1/2*. Multiple gene panel testing on moderate- to low-penetrance genes identified those who have previously tested negative in genetic tests [26]. Current NCCN germline testing has been mainly restricted to high-risk predisposition genes, where classification and management guidelines are better defined. The answer to the question of whether to expand germline genetic testing to include a panel not limited to the 6 listed high-penetrance genes in NCCN testing guidelines depends on the determination by local and regional healthcare institutions and their policymakers [27]. However, there are still little available odds ratio data regarding the cancer risk for the low-penetrance genes and their specific guidelines for risk management [28,29]. This issue is augmented by the low incidence rates of hereditary disease, leading to difficulty conducting large cohort representation studies [30].

We identified 242 patients who met the 2023 v.1 criteria only but not the 2022 v.2 criteria. The mutation pick-up rate on the 30-gene panel was 2% for patients with bilateral breast cancer. In another study on 139 bilateral breast cancer patients of unselected age, the overall mutation rate was 37.4%. The mutation rates on *BRCA1/2* and the 6 high-penetrance genes were 23.7% and 30.2%, respectively [31]. Over 67% of these patients were diagnosed before 50. Our low mutation rate of 2% reflected the late-onset patients diagnosed with bilateral breast cancer (diagnosed after age 50) and with no family history of hereditary-related cancers. This mutation rate was even lower than in a study on random consecutive breast cancer samplings in Chinese. The *BRCA1/2* mutation rates in this consecutive random cohort were 5.3% and 6.5% from 6 high-penetrance genes [32]. Our latest pick-up rates on patients with bilateral breast cancers with unselected age and family history were 21%, 16% from *BRCA1/2* mutations, and 19% from 6 high-penetrance genes (data from out rountine lab).

In our study cohort, 94 (2.5%) patients did not meet both testing criteria, and the mutation rates for these patients were 5.3% for *BRCA1/2*, 6.4% in all 6 high-penetrance genes, and 8.5% for the 30-gene panel. In this cohort, 24 (25.5%) of these patients were recruited because of their personal history of multiple cancers, and 1 (4.2%) *BRCA1* mutation carrier was identified. Three patients were tested because of patient intention; 2 out of 3 (66.7%) were *BRCA1* mutation carriers. Other unclassified cases that met neither criteria were recruited at the startup of the registry with less stringent entry requirements or information only recorded by patient verbal descriptions.

The mutation detection rate after the relaxation of the NCCN testing criteria was reviewed in the Chinese population. An extra 3.7% (9 out of 242) of the patients with mutations in 30 hereditary-cancer-associated genes will benefit. Starting from early 2014, ACMG and individual research groups suggested that all patients with breast cancer should be evaluated for the need to have germline genetic testing for hereditary breast cancer [12,13,14]. In recent years, a Mayo Clinic study proposed a hybrid approach of testing all women diagnosed with breast cancer by the age of 65 years while using the NCCN criteria for older patients [33]. However, there are still many limitations on these test approaches. A low positive prediction value on all breast cancer patients and the lack of evidence base input for rarer genes to support risk management included in many multigene panels were always a consideration between the balance of resources. Shortage of well-trained genetics counselor and health professionals was happening worldwide, which requires further consideration.

## 5. Conclusions

We provided a review in our Hong Kong high-risk breast cancer cohort to evaluate the increase in allocated resources during the paths of expansions in mutation carriers identified by multigene panel testing compared with those included in the current risk-stratified approach NCCN eligibility criteria, before the test was performed on all breast cancer patients. Whether it is worth spending the resources on testing all patients depends on the clinical characteristics and resource limitations, such as providing surveillance cancer screenings, prophylactic surgeries and caring, genetics counseling, and reproductive planning support, not only to these patients but also to their family members.

## Figures and Tables

**Table 1:** Clinicopathological characteristics of patients recruited.

		*n* = 3797	%
Sex	F	3713	97.8%
	M	84	2.2%
First diagnosis age	Mean	45.5	(SD) 11.5
	Median	44	(Range) 18–95
Personal multiple cancers		305	8.0%
Bilateral breast		747	19.7%
Pathology (primary tumors = 4544)			
Histology	Ductal	3212	72.7%
	In situ	721	16.3%
	Others	485	11.0%
	NS	126	
Stage	0	775	18.1%
	I	1573	36.8%
	II	1307	30.5%
	III	471	11.0%
	IV	154	3.6%
	Not stated	264	
Grade (invasive)	1	564	17.9%
	2	1437	45.7%
	3	1146	36.4%
	Not stated	676	
Breast cancer subtype	Luminal type	2699	75.5%
	TNBC	573	15.8%
	HER2+	305	8.5%
	Not stated	246	
Family history in 1st–3rd degrees	Breast cancer	1655	43.6%
	Ovarian cancer	202	5.3%
	Prostate cancer	201	5.3%
	Pancreatic cancer	186	4.9%

**Table 2:** Major changes in testing criteria on NCCN 2022 v2 and 2023 v1.

		Proband’s Breast Cancer Diagnosis Age	
Testing Criteria		2022 v2	2023 v1
Personal breast cancer	Diagnosis age	≤45 y	≤50 y
	Multiple primary breast cancers (Synchronous or metachronous)	46–50 y	Any age
Family history (≥1 close relative ^ with)	Breast cancer at any age	46–50 y	-
	Breast cancer at age ≤50 y	≥51 y	Any age
	Male breast cancer at any age	≥51 y	Any age
	Ovarian, pancreatic, or metastatic/high-risk group of prostate cancer at any age	46–50 y	Any age
Family history (≥2 close relative ^ with)	Breast or prostate cancer at any age	≥51 y	Any age
Family history (≥3 in patient and/or close relative ^ with)	Breast cancer at any age	≥51 y	Any age

^ First, second, and third degrees’ family history.

**Table 3:** Mutation rate in NCCN 2022 v.2 and 2023 v.1 criteria.

	Mutation Positive			Negative	Grand Total
	*BRCA1/2*+	6 Gene+	30 Genes+		
Total recruited probands	361 (9.5%)	435 (11.5%)	514 (13.5%)	3283	3797
Meeting 2022 v2 criteria	351 (10.1%)	423 (12.2%)	497 (14.4%)	2964	3461 (91.2%)
Meeting 2023 v1 criteria	356 (9.6%)	429 (11.6%)	506 (13.7%)	3197	3703 (97.5%)
Meeting 2023 v1 criteria only but not 2022 v.2	5 (2.1%)	6 (2.5%)	9 (3.7%)	233	242 (6.4%)
Not meeting both criteria	5 (5.3%)	6 (6.4%)	8 (8.5%)	86	94 (2.5%)

**Table 4:** Clinicopathological characteristics of 242 patients who met 2023 v1 criteria only but not 2022 v.2.

		Mutation Positive			Negative	Total	*p*-Value (30 Genes + vs. Negative)
		*BRCA1/2*+	6 Gene+	30 Genes+			
		*n* = 5	*n* = 6	*n* = 9	*n* = 233	*n* = 242	
Diagnosis age	Mean	51.23	50.58	50.83	56.71	56.49	0.006
	Median	50.56	49.3	48.03	53.97	53.65	0.058
	Range	48–56	47–56	46–59	46–84	46–84	
	≤50	3 (3.6%)	4 (4.8%)	6 (7.1%)	78 (92.9%)	84 (34.7%)	0.0681
Bilateral		2 (1.3%)	2 (1.3%)	3 (2%)	147 (98%)	150 (62%)	0.0873
Histology	Ductal	4 (1.6%)	5 (2%)	7 (2.8%)	243 (97.2%)	250 (63.8%)	0.1066
	In situ	1 (1.1%)	1 (1.1%)	3 (3.3%)	88 (96.7%)	91 (23.2%)	
	Others	0	0	0	38 (100%)	38 (9.7%)	
	Not stated	2	2	2	11	13 (3.3%)	
Breast cancer subtype	Luminal type	6 (2.6%)	7 (3%)	9 (3.9%)	223 (96.1%)	232 (59.2%)	0.5886
	TNBC	0	0	0	6 (100%)	6 (1.5%)	
	HER2+	0	0	0	46 (100%)	46 (100%)	
	Not stated	0	0	0	17	17 (4.3%)	
Grade (invasive)	Low/intermediate	2 (1.1%)	2 (1.1%)	4 (2.1%)	183 (97.9%)	187 (47.7%)	0.3654
	High	2 (3.3%)	3 (5%)	3 (5%)	57 (95%)	60 (15.3%)	
	Not stated	2	3	2	52	54 (13.8%)	
Stage of Breast	0	1 (1%)	1 (1%)	3 (3.1%)	93 (96.9%)	96 (24.4%)	0.8773
	I	5 (4%)	5 (4%)	6 (4.8%)	120 (95.2%)	126 (32.1%)	
	II	1 (1.1%)	1 (1.1%)	2 (2.2%)	87 (97.8%)	89 (22.7%)	
	III	0	1 (2%)	1 (2%)	48 (98%)	49 (12.5%)	
	IV	0	0	0	12 (100%)	12 (3.1%)	
	Not stated	0	0	0	20	20 (5.1%)	

## Data Availability

The dataset supporting the conclusions of this article is included within the article and its additional files.

## Supplementary Materials

The following supporting information can be downloaded at: https://www.mdpi.com/article/10.3390/cancers15092635/s1, Table S1: 30 Genes Panel covering most of the relevant genes for mutations that could increase risk for Breast, Ovarian, Uterine, Colorectal, Melanoma, Pancreatic, Stomach, Prostate.
