# Supplementary material for: Effect on Germline Mutation Rate in a High-Risk Chinese Breast Cancer Cohort after Compliance with The National Comprehensive Cancer Network (NCCN) 2023 v.1 Testing Criteria
Source: Cancers (Basel). 2023 May 6;15(9):2635. doi: 10.3390/cancers15092635 (PMC10177488; doi:10.3390/cancers15092635)
Supplement: Supplementary file 1 [file cancers-15-02635-s001.zip › cancers-2325826-supplementary.pdf]

Supplementary Table S1. 30 Genes Panel covering most of the relevant genes for mutations that could increase risk for Breast, Ovarian, Uterine, Colorectal, Melanoma, Pancreatic, Stomach, Prostate

**30 Genes Panel**					
BRCA1	BRCA2	MLH1	MSH2	MSH6	PMS2***
EPCAM**	APC	MUTYH	MITF**	BAP1	CDKN2A
CDK4**	TP53	PTEN	STK11	CDH1	BMPR1A
SMAD4	GREM1**	POLD1**	POLE**	PALB2	CHEK2
ATM	NBN	BARD1	BRIP1	RAD51C	RAD51D

** Only positions known to impact cancer risk analyzed: CDK4: only chr12:g.58145429-58145431 (codon 24) analyzed, EPCAM: only large deletions and duplications including 3' end of the gene analyzed, GREM1: only duplications in the upstream regulatory region analyzed, MITF: only chr3:g.70014091 (including c.952G>A) analyzed, POLD1: only chr19:g.50909713 (including c.1433G>A) analyzed, POLE: only chr12:g.133250250 (including c.1270C>G) analyzed.

*** PMS2: Exons 12-15 not analyzed.
